# Diffuse Leptomeningeal Glioneuronal Tumor: First Description of Metastasis to the Lung and Bone Marrow

**DOI:** 10.7759/cureus.48185

**Published:** 2023-11-02

**Authors:** Sahithi Battini, Murat Gokden, Viktoras Palys, Erika Santos Horta

**Affiliations:** 1 Department of Neurology, University of Arkansas for Medical Sciences, Little Rock, USA; 2 Department of Pathology, University of Arkansas for Medical Sciences, Little Rock, USA; 3 Department of Neurosurgery, University of Arkansas for Medical Sciences, Little Rock, USA; 4 Winthrop P. Rockefeller Cancer Institute, University of Arkansas for Medical Sciences, Little Rock, USA

**Keywords:** extra cranial metastasis, neuro-oncology, primary brain tumor, leptomeningeal carcinomatosis, diffuse leptomeningeal glioneuronal tumor, metastatic disease

## Abstract

Diffuse leptomeningeal glioneuronal tumor (DLGNT) is a rare neoplasm of the central nervous system (CNS) that primarily affects the leptomeninges. However, it can also involve the brain parenchyma and spinal cord. We report the first case of metastasis of this primary CNS tumor to the lung and bone marrow. An 18-year-old male was diagnosed with DLGNT through meningeal biopsy after multiple events of transient neurologic signs and symptoms that included recurrent episodes of encephalopathy, seizures, cerebral vasospasms, cranial nerve palsy, and urinary dysfunction. Five months after diagnosis, the patient presented with pancytopenia and pulmonary effusion. At that time, he was being treated with temozolomide, after radiation treatment to the brain and spinal cord. Bone marrow biopsy and pleural cytology revealed systemic metastases from the primary CNS tumor. He was then treated with chemotherapy with carboplatin and vincristine which improved his condition for two and a half months. Unfortunately, the patient died of a high systemic metastatic burden. Primary CNS tumors rarely produce systemic metastases, and this is the first report of DLGNT with bone marrow and pulmonary metastases. Chemotherapy with carboplatin and vincristine should be considered as a treatment for patients with DLGNT, as the patient presented a systemic response with clinical and radiological improvement.

## Introduction

Diffuse leptomeningeal glioneuronal tumor (DLGNT) is a rare primary central nervous system (CNS) neoplasm, the incidence of which is unknown [1]. It was first added to the World Health Organization (WHO) classification in 2016 [2]. Epidemiologically, more males than females are diagnosed with this tumor [1,2]. It is more common in children, but there are also cases in adults [1-3]. The tumor usually spreads in the leptomeninges but can also be associated with mass-like lesions in the spine or brain parenchyma [1-4]. Clinical complications include hydrocephalus, seizures, paresis, and hemiplegic migraine, among other symptoms [3,5-8]. Overall survivorship can vary from three months to 21 years [1].

Microscopically, DLGNT is characterized by oligodendroglial-like cells with evidence of neuronal differentiation [1,2]. With the rise in the importance of molecular pathology in neuro-oncology, further characterization is possible [2,9-11].

DLGNT is an isocitrate dehydrogenase (IDH)-wildtype, with a 1p chromosomal deletion tumor [1,2,12]. KIAA1549-BRAF fusion is identified in 60-75% of cases, and BRAF V600E mutation in about 10% [9,11-13]. In 2021, WHO updated the DLGNT definition, incorporating diagnostic molecular alterations and identified subtypes classified by different methylation profiles [2,9]. Regarding prognosis, a Ki-67 proliferation index higher than 4-7%, age over nine years old, elevated intracranial pressure, and subtype 2 methylation pattern have been proposed as risk factors for worse prognosis in children [12,14]. In contrast, patients presenting KIAA 1549-BRAF fusion might have a better outcome [12].

Due to the rarity of this diagnosis, there are no clinical trials to guide therapy. A literature review identified several treatment options, including temozolomide, combined carboplatin and vincristine, and targeted therapy like BRAF and MEK inhibitors [7,9,12,15,16]. 

This article was previously presented as a meeting abstract at the 2022 Society of Neuro-Oncology Conference on November 17, 2022, in Tampa Bay, Florida, United States.

## Case presentation

An 18-year-old male with a history of hereditary multiple osteochondromas was initially admitted for investigation of confusion, temporal headache, unsteady gait, and left hemiparesis after two months of symptomatic coronavirus disease 2019 (COVID-19) infection. Computed tomography angiography (CTA) performed at another institution was reported concerning for possible vasospasm in the right internal carotid artery, right anterior communicating artery, and right middle cerebral artery; there were no restricted-diffusion lesions upon brain magnetic resonance imaging (MRI). At that time, lumbar puncture showed high protein levels (299 milligrams per deciliter) and a mild increase in cells (9 WBC per microliter), with macrophage predominance. Four-vessel angiogram done days after the CTA was unremarkable. The patient's symptoms spontaneously resolved, and he was diagnosed with possible reversible cerebral vasoconstriction syndrome and was discharged on verapamil and magnesium.

Two weeks later, he developed acute onset right-sided weakness and aphasia, and the clinical findings were similar to those of the previous episode. CTA again indicated vasospasm of the left middle cerebral artery, but there were no acute findings with MRI. A new lumbar puncture was repeated with an increase of protein (501 milligrams per deciliter), and an increase in the number of cells (17 WBC per microliter) with monocytic predominance, and normal glucose. The infectious panel was negative and the angiotensin-converting enzyme was mildly elevated (2.8 U/L). Again, a four-vessel angiogram was normal. Video electroencephalogram (EEG) showed slowing in the left frontotemporal region and generalized slowing. The patient's neurological deficits improved and he was discharged with the diagnosis of probable headache and neurological deficits with cerebrospinal fluid lymphocytosis (HaNDL syndrome).

Ten days later, the patient was admitted again with headache, confusion, and brisk reflexes. EEG showed left hemispheric (mainly frontotemporal) slowing and generalized slowing. Overnight, the patient developed left eye ptosis with mydriasis, right eye miosis, left-sided weakness, and drowsiness. Treatment with intravenous high-dose methylprednisolone (1000 mg/day for five days) was started without improvement. The patient progressed with diplopia and sixth nerve palsy when ophthalmology was consulted. MRI brain was repeated and did not show any lesions.

**Figure 1:**
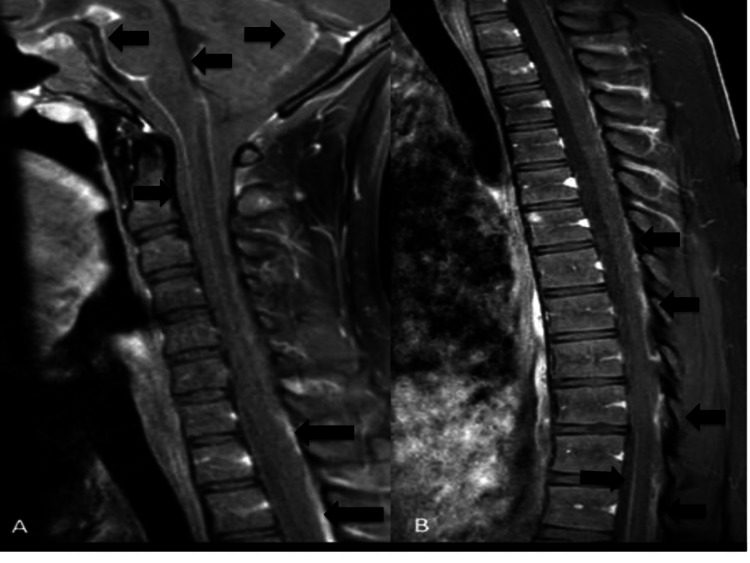
T1-weighted post-contrast MRI of cervical (A) and thoracic (B) spine showing leptomeningeal enhancement

Two weeks later, he presented with urinary retention and a possible seizure. Treatment with intravenous high-dose methylprednisolone was repeated and levetiracetam was added. EEG showed moderate generalized slowing. This time, brain MRI showed suspected basal meningitis, acute infarcts in the posterior body, genu of the corpus callosum and left cerebral hemisphere along the watershed zone, and mild interval increase in the size of ventricles. Spine MRI revealed meningeal enhancement along the upper cervicothoracic spinal cord and enhancement of cauda equina nerve roots (Figure 1).

**Figure 2:**
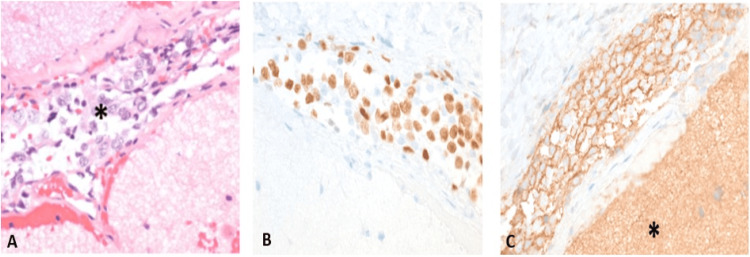
Microscopic pathology from leptomeningeal biopsy (original magnifications: 400x) (A) Leptomeningeal infiltration by neoplastic cells (*), sparing the adjacent neuroglial parenchyma; (B) Immunohistochemical positivity for olig2, a glial marker; (C) CD57, a neural marker, which is also normally positive in the brain parenchyma

At that moment, three months from the first symptoms, a meningeal biopsy was performed. Pathology described a leptomeningeal neoplastic infiltrate with cells positive for S-100 protein, SOX10, CD56, CD57, Olig2, and negative for MART-1, HMB-45, CD99, pointing to the diagnosis of DLGNT (Figure 2). Ki-67 was positive in many cells; however, accurate counting for the proliferation index could not be performed due to the presence of only a small number of cells, but it was considered to be high. Unfortunately, his seizures worsened. Upon investigation, CT of the brain showed hydrocephalus, and a ventriculoperitoneal shunt was implanted using a programmable vase (Polaris® SPVA (without anti-siphon device), Sophysa SA, Orsay, Ile-de-France, France) and laparoscopic assistance. 

**Figure 3:**
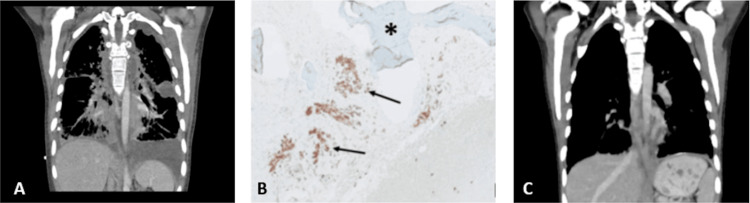
Pulmonary and bone marrow metastases (A) CT Chest with diffuse nodular parenchymal disease and bilateral pleural effusions; (B) Bone marrow biopsy showing  bone trabeculae (*) and cells that are positive for Olig2 (arrows); (C) CT Chest with an improvement of nodular parenchymal disease and improvement of bilateral pleural effusions

Three weeks after his meningeal biopsy, he began treatment with CNS radiation therapy (six weeks) concurrent with temozolomide (75 milligrams per square meter). Unfortunately, temozolomide was withheld during the first weeks of the radiation therapy due to pancytopenia. Four weeks after finishing radiation therapy, temozolomide 150 mg per square meter was resumed for five days every 28 days. After his second cycle, the patient presented with pancytopenia that did not improve after withholding chemotherapy. A bone marrow biopsy was then performed. One week after the procedure, the patient presented with dyspnea and needed supplementary oxygen. On investigation, bilateral pleural effusions with diffuse nodular parenchymal disease were seen on chest CT (Figure 3A) Both bone marrow biopsy (Figure 3B) and pleural liquid were positive for DLGNT. A liquid biopsy did not reveal a targetable mutation. The patient began treatment with carboplatin (175 milligrams per square meter) weekly for four weeks every six weeks and vincristine (2 mg) weekly for 10 weeks. Lung infiltrates and pancytopenia improved after three weeks; his symptoms improved, and the patient no longer needed supplementary oxygen or transfusion (Figure 3C). Unfortunately, 10 weeks later, the patient noticed weakness in his face, and metastatic disease to the muscles of the face was identified on brain MRI. The patient passed away the next month, one year and one month after his first presentation.

## Discussion

DLGNT is an extremely rare primary CNS tumor, and there are no treatment guidelines for this condition. Knowledge regarding treatment is based on retrospective studies, case reports, series of cases, and systematic reviews, but there have been no clinical trials. Multiple options of chemotherapy are reported in mono- or combination therapy including temozolomide, carboplatin, vincristine, cisplatin, etoposide, procarbazine, lomustine, and targeted therapy with MEK inhibitor [5-7,11,12,16].

DLGNT can often be difficult to diagnose because patients experience a broad spectrum of symptoms, including hemicrania migraine, other recurrent headaches, hydrocephalus, cranial nerves dysfunction, seizures, gait disorder, aphasia, and urinary dysfunction [3,5-8,11,12,16]. The patient, in the current case, sought medical care four times with different symptoms before he was correctly diagnosed. The literature describes that patients undergo an extensive infectious and rheumatological workup or are diagnosed and treated for other conditions until a meningeal or brain biopsy is done [4,5]. Therefore, differential diagnosis can include CNS tuberculosis and other infections meningitis, neurosarcoidosis, hemiplegic migraine, disseminated systemic cancer, and HaNDL syndrome [3-6,8]. Nevertheless, the question of how to diagnose this condition at the beginning of the symptoms persists. For that, it is important to raise awareness of this diagnosis not only for pediatric and adult neurologists, but for all specialties including emergency department specialists, oncologists, pathologists, and neurosurgeons. Currently, the diagnosis is tissue-based [1,2], and therefore meningeal and/or CNS biopsy is imperative to be done as soon as possible. In the future, the diagnosis through cell-free DNA in the CSF might be a possibility, as in a study of seven patients, this analysis showed to have high diagnostic sensitivity [10,17].

We considered whether placing the ventriculoperitoneal shunt predisposed the patient to systemic metastases. This is unlikely, because about 20-25% of patients with DLGNT require a ventriculoperitoneal shunt as treatment of hydrocephalus, but there are no previous descriptions of systemic metastases [12]. If the ventriculoperitoneal shunt was the source of systemic metastases, the peritoneum would be the first location of metastases. In this particular patient, the peritoneum was not examined pathologically due to late notification of death that occurred as an outpatient in another city, not allowing an autopsy to be requested by the treating team. Even so, we do not have any evidence to suspect that there were peritoneal metastases, as the patient underwent a CT abdomen without any lesions, and never presented ascites or any abdominal symptoms. Although no clinical trials have been conducted, multiple studies, including systematic reviews and meta-analyses consider ventriculoperitoneal shunt and other CSF diversion techniques to treat hydrocephalus in patients with leptomeningeal disease due to systemic cancer [18-20]. Although multiple complications can occur in ventriculoperitoneal shunts in patients with leptomeningeal disease (from a systemic cancer like breast or lung, or primary brain tumors like gliomas and medulloblastoma), new or worsening metastases are not common complications of the procedure [18-20]. In fact, ventriculoperitoneal shunts can improve symptoms and can prolong overall survivorship in patients with hydrocephalus due to leptomeningeal carcinomatosis [18-20]. 

This case study is the first description of metastases of DLGNT outside the CNS. Here, the patient presented with symptomatic metastatic disease of the lungs and bone marrow after treatment with temozolomide and radiation therapy. It is important to mention that this patient had numerous risk factors for poor prognosis related to DLGNT: high Ki-67 proliferation index for tumor cells, >nine years old, elevated intracranial pressure requiring a ventriculoperitoneal shunt, and no KIAA-BRAF fusion mutation in tumor cells [9,11]. We did not have sufficient tumor samples to test the methylation profile to determine if the tumor was subtype 1 or 2. In this case, MEK inhibitors were not prescribed because no targetable mutations were identified in the liquid biopsy performed at the time that metastases were identified, and the sample from his meningeal biopsy lacked sufficient material for next-generation sequencing. Notably, the patient experienced an improvement in disease burden after treatment with vincristine and carboplatin was initiated, suggesting that this treatment should be considered for metastatic disease. 

## Conclusions

DLGNT is a rare primary CNS tumor recently added to the WHO classification of brain tumors. Research has greatly advanced knowledge regarding DLGNT pathology, molecular characterization, and possible risk factors for poor prognosis. Unfortunately, due to the rarity of the tumor and the lack of clinical trials, there is no clear treatment guideline.

Here, we add to the clinical knowledge on DLGNT by reporting that this primary brain tumor can produce systemic metastases. This report also notes clinical and radiological improvement of DLGNT metastasis upon chemotherapy with carboplatin and vincristine. Therefore, the combination of carboplatin and vincristine should be considered as a treatment for patients with DLGNT.
